# Nowcasting (Short-Term Forecasting) of COVID-19 Hospitalizations Using Syndromic Healthcare Data, Sweden, 2020

**DOI:** 10.3201/eid2803.210267

**Published:** 2022-03

**Authors:** Armin Spreco, Anna Jöud, Olle Eriksson, Kristian Soltesz, Reidar Källström, Örjan Dahlström, Henrik Eriksson, Joakim Ekberg, Carl-Oscar Jonson, Carl-Johan Fraenkel, Torbjörn Lundh, Philip Gerlee, Fredrik Gustafsson, Toomas Timpka

**Affiliations:** Linköping University, Linköping, Sweden (A. Spreco, O. Eriksson, R. Källström, Ö. Dahlström, H. Eriksson J. Ekberg, C.-O. Jonson, F. Gustafsson, T. Timpka);; Region Östergötland, Linköping (A. Spreco, R. Källström, J. Ekberg, C.-O. Jonson, T. Timpka);; Lund University, Lund, Sweden (A. Jöud, K. Soltesz);; Skåne University Hospital, Lund (A. Jöud. C.-J. Fraenkel);; Chalmers University of Technology, Gothenburg, Sweden (T. Lundh, P. Gerlee);; Gothenburg University, Gothenburg (T. Lundh, P. Gerlee)

**Keywords:** COVID-19, coronavirus disease, SARS-CoV-2, severe acute respiratory syndrome coronavirus 2, viruses, respiratory infections, zoonoses, nowcasting, forecasting, infectious diseases, epidemiology, public health, medical informatics, Sweden, *Suggested citation for this article*: Spreco A, Jöud A, Eriksson O, Soltesz K, Källström R, Dahlström Ö, et al. Nowcasting (short-term forecasting) of COVID-19 hospitalizations using syndromic healthcare data, Sweden, 2020. Emerg Infect Dis. 2022 Mar [*date cited*]. https://doi.org/10.3201/eid2803.210267

## Abstract

We report on local nowcasting (short-term forecasting) of coronavirus disease (COVID-19) hospitalizations based on syndromic (symptom) data recorded in regular healthcare routines in Östergötland County (population ≈465,000), Sweden, early in the pandemic, when broad laboratory testing was unavailable. Daily nowcasts were supplied to the local healthcare management based on analyses of the time lag between telenursing calls with the chief complaints (cough by adult or fever by adult) and COVID-19 hospitalization. The complaint cough by adult showed satisfactory performance (Pearson correlation coefficient *r*>0.80; mean absolute percentage error <20%) in nowcasting the incidence of daily COVID-19 hospitalizations 14 days in advance until the incidence decreased to <1.5/100,000 population, whereas the corresponding performance for fever by adult was unsatisfactory. Our results support local nowcasting of hospitalizations on the basis of symptom data recorded in routine healthcare during the initial stage of a pandemic.

During the initial stage of the coronavirus disease (COVID-19) pandemic, large variations in virus dissemination within countries often led to lack of sufficiently specific information for local authorities to make accurate decisions about health service adjustments (*1*,*2*). The situation was further worsened by heterogeneity in virus testing strategies, usually a result of local differences in laboratory capacities (*3*), leading to a need for local-scale COVID-19 forecasting methods based on resources available in the existing healthcare infrastructure (*4*). In particular, experts called for short-term forecasts of incident hospitalizations to plan staff reallocation and creation of temporary facilities for intensive or subintensive care with ventilators (*5*).

We have previously developed a local influenza nowcasting (short-term forecasting) method whereby syndromic healthcare data are used to nowcast later diagnostic events (*6*). The method has shown satisfactory performance in prospective evaluations (*7*,*8*). We used this experience during the initial stage of the pandemic in 2020 to nowcast local cases of patients hospitalized with COVID-19 by modeling associations with data from Swedish Healthcare Direct’s 24-hour telenursing service (telephone number 1177) (*9*). Telenursing services are available in numerous countries for health counseling and evaluation of clinical service needs in the general population (*10*–*12*). In Sweden, the chief complaint for each call is recorded in an administrative database (*13*). During the 2009 influenza pandemic, records of telenursing chief complaints were used to forecast variations in local healthcare load, although less accurately than during regular influenza seasons (*14*).

The purpose of our study was to examine the performance of syndromic healthcare data in nowcasting local hospital admissions during the initial stage of the COVID-19 pandemic, when resources for diagnostic laboratory testing were limited. The specific aim was to investigate the prospective performance of symptoms recorded during telenursing calls in nowcasting daily cases of patients hospitalized with COVID-19 during March–June 2020 in Östergötland County, Sweden (population ≈465,000). The Swedish Ethical Review Authority (dnr. 2020-03183) approved the study design. Because COVID-19 and influenza share characteristic symptoms, we interpreted the performance of the COVID-19 nowcasting using syndromic symptom data, taking into consideration parallel winter influenza activity in the county. 

## Methods

We used prospective evaluation design; that is, we defined the COVID-19 nowcasting procedure and the evaluation protocol before beginning to collect evaluation data. The management of Region Östergötland, the public (tax-financed) healthcare provider serving Östergötland County, used the daily nowcasts we created for planning resource allocation. Nowcasting of COVID-19 hospitalizations was based on the time lag from telenursing calls with selected chief complaints (Appendix); we retrieved nowcasting data from the countywide health information system managed by the healthcare provider (*15*). Because the COVID-19 pandemic reached the study county during an ongoing influenza season, we describe the progress of both local epidemics for comparison.

### Data Sources

Syndromic data were recorded from telenursing calls made by county residents to Swedish Healthcare Direct. Daily numbers of calls with chief complaints possibly associated with COVID-19 were retrieved from Hälsoläge, the national database, using the fixed-field terminology register service (*16*). The diagnostic data were collected from patients hospitalized with the International Classification of Diseases, 10th Revision (ICD-10), code U07.1 (COVID-19, virus identified). All patients hospitalized with suspected COVID-19 were given a PCR test for virus identification and diagnosis.

We retrieved daily numbers of patients diagnosed with laboratory-confirmed influenza (inpatient and outpatient) for February 20–June 30, 2020. For comparison, we also retrieved corresponding influenza and telenursing chief complaint data for the same period for each year during 2015–2019.

### Nowcasting Procedure

We began developing the local COVID-19 nowcasting procedure on February 20, 2020. During March 2–6, we examined peer-reviewed scientific reports on COVID-19 symptoms to select telenursing chief complaints for the nowcasting, (*17**–**19*). The largest study retrieved, involving 1,099 patients from 30 provinces in China, reported fever (89%) and cough (68%) to be the most common symptoms, followed by fatigue (38%), shortness of breath (19%), and sore throat (14%) (*17*). The study also reported that hospitalized patients were almost exclusively adults. In the selection of corresponding telenursing chief complaints for use in nowcasting, we excluded unspecific symptoms of upper respiratory tract infection (fatigue and sore throat) and complaints expected to lead to a recommendation for immediate physical examination (shortness of breath). We chose the remaining telenursing chief complaints, cough by adult and fever by adult, as syndromic variables for use in the nowcasting of COVID-19 hospitalizations. We finalized the procedure on March 20.

#### Definition of Time Lag

After consultations with local healthcare managers, we found that we needed short-term forecasts in the interval of 14–21 days for implementing adjustments of hospital resources. To select the time lag in the interval with the highest correlation (i.e. the highest Pearson correlation coefficient, *r*) between syndromic and hospital admission data, we performed analyses of time series data from the previous 4 weeks for each of the 2 syndromic variables, leading to 16 possible outcomes: 8 time lags of 14–21 days for each variable. To eliminate weekday effects, we smoothed all series by calculating a 7-day moving average. If correlations for time lags were equal, we chose the longest. To adjust for the higher daily numbers of telenursing calls compared with hospitalization cases, we multiplied the level for each of the 2 chief telenursing complaints by a ratio calculated by dividing the sum of hospitalizations during a 14-day period by the sum of telenursing calls (separately for each syndromic variable) over a previous 14-day interval at a time distance, chosen depending on the resulting best time lag. The length of the interval should be a multiple of 7 days to level out weekday effects and be about the same as the time lag. Therefore, we chose an interval of 14 days.

#### Hospital Admission Nowcasting

We created daily nowcasts and forwarded them to the healthcare management at Region Östergötland beginning March 22, 2020. We performed a new calculation of the correlation coefficient each nowcasting day and chose the time lag with the highest correlation for each of the 2 chief complaints for nowcasts. We performed daily nowcasts of forthcoming hospitalizations for the period covered by the time lag between COVID-19 hospitalizations and telenursing calls for cough by adult and fever by adult throughout the study period (Appendix).

### Descriptive Analyses

Because COVID-19 and influenza share symptoms (telenursing chief complaints), we examined the daily numbers of COVID-19 hospitalizations and cases of laboratory-confirmed influenza in Östergötland County (primary and hospital care) for the period February 20–June 30, 2020. We also descriptively analyzed the annual trends for this period in 2015–2019 for cases of laboratory-confirmed influenza and for the telenursing chief complaints cough by adult and fever by adult.

### Evaluation Procedure was defined

We evaluated the nowcasting performance during March 22–June 30, 2020. We defined the evaluation protocol on March 20 and followed it without alteration throughout the evaluation period. We evaluated performance by calculating the correlation between trends in the selected telenursing calls and trends in later hospitalizations, and by determining the accuracy of the nowcasted incidence of daily hospitalizations. The outcome measures were the Pearson correlation coefficient between the telenursing and hospitalization data from the nowcasting date through the period covered by the time lag (denoted as *r*^FND^) and the mean absolute percentage error (MAPE) of the nowcasted hospitalization incidence. *r*^FND^ can vary between −1 and 1 (where −1 is perfect negative correlation and 1 is perfect positive correlation). The lower limit for MAPE is 0; an upper limit does not exist. Before beginning data collection, we defined the limits for satisfactory nowcasting performance as *r*^FND^ >0.80 and MAPE <20%.We derived the limit for *r*^FND^ from previous nowcasting studies (*20*) and determined the MAPE limit, following discussions with health service managers, on the basis of hospital resources in Sweden, which were overextended before the COVID-19 pandemic (on average, 103 patients occupied 100 administrative hospital bed units [*21*]).

## Results

### COVID-19 Pandemic

**Figure 1:**
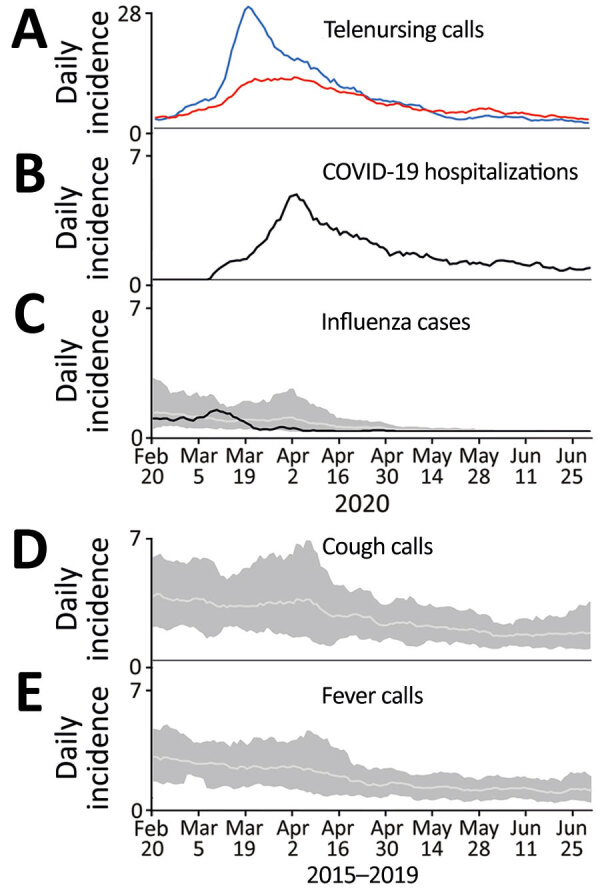
Daily incidence of telenursing calls for 2 chief complaints, COVID-19 hospitalizations, and laboratory-confirmed influenza plus reference data from before the COVID-19 pandemic, Östergötland County, Sweden. A) Telenursing calls per 100,000 population for chief complaints of cough by adult (blue line) and fever by adult (red line), February 20–June 30, 2020. B) COVID-19 hospitalizations per 100,000 population, February 20–June 30, 2020. C) Cases of laboratory-confirmed influenza per 100,000 population February 20–June 30, 2020 (black line). Light gray line indicates the average for cases of laboratory-confirmed influenza in 2015–2019; dark gray shaded area is the corresponding range. D) Telenursing calls per 100,000 population for the chief complaint cough by adult in 2015–2019 (light grey line) with corresponding range (dark grey shaded area). E) Telenursing calls per 100,000 population for the chief complaint fever by adult in 2015–2019 (light grey line) with corresponding range (dark grey shaded area).

Calls by Östergötland county residents to Swedish Healthcare Direct with the chief complaint of cough by adult peaked on March 21 (Figure 1, panel A). On the same day, calls for the complaint fever by adult reached a plateau that lasted for ≈2 weeks (until April 3) (Figure 1, panel A).

The first hospitalization in Östergötland County for COVID-19 occurred on March 8, 2020. At the start of the evaluation period on March 22, the daily hospitalization incidence was 1.8 patients/100,000 population; peak incidence (4.9 patients/day/100,000 population) was reached on April 2 (Table; Figure 1, panel B). In mid-May, the daily incidence had declined to <1.5 hospitalizations/100,000 population; it was 0.6 hospitalizations/100,000 population on June 30, the end of the study period.

**Table:** Weekly nowcasting performance for 2 syndromic variables in the first wave of the coronavirus pandemic, Östergötland County, Sweden, 2020*

Nowcasting dates	Hospitalizations/day/100,000 population	Cough by adult			Fever by adult	
		*r* ^FND^	MAPE		*r* ^FND^	MAPE
Week 1 (Mar 22–28)	1.8–3.4	0.86–0.97	9 –28		0.01–0.99	14–20
Week 2 (Mar 29–Apr 4)	3.4–4.9	0.93–0.98	3–5		−0.63 to −0.32	17–47
Week 3 (Apr 5–11)†	3.2–4.5	0.89–0.95	4–6		−0.20 to 0.79	39–52
Week 4 (Apr 12–18)	2.6–3.2	0.92–0.97	4–6		0.87–0.95	16–45
Week 5 (Apr 19–25)	2.1–2.6	0.74–0.94	6–9		0.70–0.93	15–21
Week 6 (Apr 26–May 2)	1.4–2.1	0.46–0.73	10–13		0.58–0.73	9–13
Week 7 (May 3–9)	1.4–1.6	0.64–0.91	7–13		0.65–0.82	8–11
Week 8 (May 10–16)	1.1–1.5	0.53–0.74	8–17		0.45–0.65	9–11
Week 9 (May 17–23)	0.9–1.1	−0.28 to 0.57	19–41		−0.08 to 0.44	9–14
Week 10 (May 24–30)	0.9–1.1	−0.87 to −0.46	38–47		−0.57 to −0.16	14–18
Week 11 (May 31–Jun 6)	0.8–1.1	−0.86 to −0.26	19–32		−0.90 to 0.63	17–28
Week 12 (Jun 7–13)	0.8–1.0	−0.03 to 0.48	29–55		0.74–0.78	21–34
Week 13 (Jun 14–20)	0.6–1.0	−0.41 to 0.36	17–48		−0.53 to 0.60	12–32
Week 14 (Jun 21–27)	0.5–0.7	−0.20 to 0.58	15–28		0.13–0.78	10–23
Week 15 (Jun 28–30)‡	0.6–0.7	0.42 to 0.50	24 to 25		0.66 to 0.70	20–22

*MAPE, mean absolute percentage error; *r*^FND^, Pearson correlation coefficient between the telenursing and hospitalization data from the nowcasting date through the period covered by the time lag. †Includes local peak of the first pandemic wave. ‡Only 3 days because it is the end of the study period.

### Influenza Season

The daily incidence of patients with laboratory-confirmed influenza peaked on March 10 (Figure 1, panel C). The recorded incidence decreased thereafter to a level that was notably below the 5-year historical trend. Calls to Swedish Healthcare Direct for the chief complaints cough by adult and fever by adult did not show a corresponding decrease in March 2020 (Figure 1, panel A). The comparative display of the historical trends from the previous 5-year period for these chief complaints showed that the levels usually increased throughout the month of March (Figure 1, panels D, E).

### Nowcasting Performance

**Figure 2:**
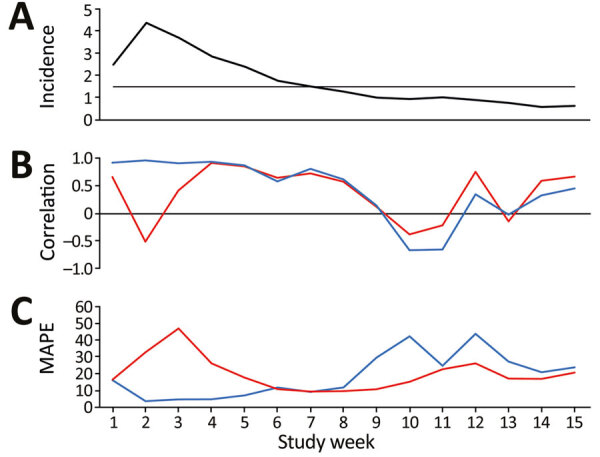
Local nowcasting performance in Östergötland County, Sweden, during the first wave of coronavirus disease (COVID-19), March 22–June 30, 2020. A) Weekly average of daily incidence of COVID-19, hospitalizations/week/100,000 population. The horizontal line indicates lowest incidence for reliable predictions (1.5 daily hospitalizations/100,000 population). B) Weekly average of daily correlation between telenursing data and COVID-19 hospitalizations from the nowcasting date through the period covered by the time lag for cough by adult (blue line) and fever by adult (red line). C) Weekly average of daily MAPE per week for cough by adult (blue line) and fever by adult (red line). MAPE, mean absolute percentage error.

The selected optimal time lag for both the cough by adult and fever by adult variables was 14 days throughout the study period, except for cough by adult during March 26–28, when the time lag was 15 or 16 days (Video). During the ascending stage of the first wave of the pandemic (March 22–April 4), as hospitalizations increased (Figure 2, panel A), *r*^FND^ for the Swedish Healthcare Direct chief complaint cough by adult was satisfactory (0.86–0.98), and MAPE decreased rapidly to a satisfactory level (from 28% to 3%) (Table; Figure 2, panels B, C; Video). *r*^FND^ for the chief complaint fever by adult decreased during this period to −0.63, and MAPE was mostly unsatisfactory (14%–47%). At the peak of the wave, with a daily hospitalization incidence >2.5/100,000 population (April 5–25), *r*^FND^ (0.74–0.97) and MAPE (4%−9%) remained satisfactory for cough by adult. For fever by adult, *r*^FND^ (−0.63 to 0.95) and MAPE (14%–52%) stayed at unsatisfactory levels. During the descending stage, *r*^FND^ and MAPE for cough by adult remained satisfactory until hospitalizations declined. When the daily hospitalizations decreased to <1.5/100,000 population in mid-May, *r*^FND^ and MAPE indicated unsatisfactory performances for both syndromic indicators (Table; Figure 2).

**Video:** Nowcasting performance using the telenursing chief complaints cough by adult and fever by adult separately during the first wave of the COVID-19 pandemic in Östergötland County, Sweden. The time series have been smoothed with a 7-day moving average to eliminate weekday effects. Black line indicates the actual number of hospitalizations per day that have already occurred when the nowcasts are calculated. Grey line indicates the actual number of hospitalizations per day that will be observed as time goes by but was not yet observed when the nowcasts were calculated. Blue line indicates the nowcasted number of hospitalizations per day based on cough by adult the following x days (where x is based on the best time lag of 14–21 days) from the day when the nowcasts are calculated. Red line indicates the nowcasted number of hospitalizations per day based on fever by adult for the following t days (where t is based on the best time lag of 14–21 days). COVID-19, coronavirus disease.

## Discussion

This study examined the performance of syndromic healthcare data (symptoms reported during telenursing calls) in nowcasting local hospital loads during the initial stage of the COVID-19 pandemic when resources for diagnostic laboratory testing were limited. We found that the telenursing chief complaint cough by adult accurately (*r*^FND^ 0.74–0.98; MAPE <10%) nowcasted local hospital loads >14 days in advance during periods with intense local dissemination of COVID-19 (corresponding to >2.5 hospitalizations/day/100,000 population) and continued to provide reliable nowcasts until the intensity decreased to <1.5 hospitalizations/day/100,000 population.

Although fever is a characteristic COVID-19 symptom, the performance of the Swedish Healthcare Direct chief complaint fever by adult in nowcasting was less satisfactory. This observation could be cause by the co-circulation of influenza virus strains and severe acute respiratory syndrome coronavirus 2 (SARS-CoV-2); fever by adult was recorded as a chief complaint from telenursing calls resulting from both influenza infection and COVID-19 (*22*). Even though cough was also a representative symptom for influenza, it appeared to be more uniquely recorded as the chief complaint from telenursing calls for COVID-19. We also observed that the incidence of patients with a laboratory-confirmed diagnosis of influenza peaked on March 10, just before the COVID-19 pandemic reached Östergötland County, and thereafter decreased to a level notably below the 5-year historical trend. It is unclear whether this decrease in the recorded incidence of influenza represents a true decline in infections or due to changes in healthcare-seeking behaviors (*23*). These observations suggest that COVID-19 nowcasting based on symptom data should be performed with caution during periods in which SARS-CoV-2 is co-circulating with influenza and other respiratory viruses.

Poor forecasting reliability during the first wave of the COVID-19 pandemic led to demands on investments in developing task-specific models and quality data collection (*24*,*25*). One explanation for the satisfactory local nowcasting performance we observed is the rapid and stable access to syndromic and diagnostic data throughout the emerging first wave of the pandemic. Most methods for COVID-19 nowcasting have used diagnostic data to model the near-future progress (typically 2–6 days) of the corresponding events (*26*); A. Altmejd, et al., unpub. data, https://arxiv.org/pdf/2006.06840.pdf). In contrast to such autoregressive models, we used a separate syndromic data source to nowcast COVID-19 hospitalizations 14–21 days in advance. This time lag to hospitalizations was needed to rearrange the local healthcare organization to care for patients with COVID-19 while minimizing collateral effects on other patient groups. We collected the syndromic and diagnostic data used for the nowcasting from a regular health information system (*15*) and analyzed the data using experiences from nowcasting the 2009 influenza pandemic and subsequent winter influenza seasons (*6*,*14*,*27*). The syndromic data were recorded by telenurses specially trained in assessment of adults and children who experienced infectious-disease symptoms (*13*). At the time of the outbreak of COVID-19 in Sweden (February 2020), telenursing had evolved from a triage practice within primary care (*28*–*31*) into a key resource in healthcare provision staffed by experienced nursing professionals (*9*). The diagnostic data we used for the nowcasting in this study were recorded using standardized coding routines (*32*) by physicians with clinical responsibility for patients hospitalized with COVID-19.

Syndromic symptom data have been used for several purposes in the early response to the COVID-19 pandemic. Using web-based data collection from the general public, the EPICOVID19 study in Italy found a strong association between olfactory and taste symptoms and laboratory-confirmed COVID-19 (*33*). Loss of smell and taste have also been reported as a characteristic COVID-19 symptom from similar research in the United Kingdom and the United States (*34*), Italy (*35*), and France (*36*). These symptom-tracking studies have provided important insights into the spectrum of COVID-19 symptoms, the rate of these symptoms in nonhospitalized persons, and the natural history of the infection. Nonetheless, for local nowcasting of hospital admissions during the early stages of a pandemic, rapid initiation of data collection and representative population coverage are required. Studies conducted in April and May 2020 showed that willingness to use a mobile application to support COVID-19 surveillance was 55%–70% in countries such as the United States, Switzerland, and Italy (*37*). However, by November 2020, the use of such mobile applications was still limited in nations where governments had promoted their development and dissemination; for example, 26% in Australia, 13% in Italy, and 2% in France (*38*). These proportions indicate that achievement of representative population coverage and continuity in data collection are challenging for COVID-19 forecasting using mobile applications. One reason for the low use of mobile applications is that legal and confidentiality issues have not been resolved for data collection from personal Internet devices in public health practice (*39*). Our nowcasting approach used trends in routinely recorded healthcare data for short-term forecasts of hospitalization cases. The approach did not require data normally unavailable for local healthcare providers and did thereby allow early initiation of nowcasting to support the local healthcare managers in their decision making.

The aim of this study was to assess hospital admission nowcasting during the early pandemic stage when broad laboratory testing still was unavailable. The syndromic variables (telenursing chief complaint codes) were thus determined in mid-March 2020 based on the information available. A limitation of the study is that it is possible that later selection of codes would have influenced the nowcasting outcomes. Also, use of individual-level telenursing data and sociodemographic data may have enabled detailed detection of municipality-level clusters during the initial stage of a pandemic. However, reports of variations in telenursing outreach and use across geographic areas and population groups, for example, among immigrants and the elderly (*12*,*40*), imply that further studies are needed to establish whether a more detailed version of our nowcasting procedure would be suitable for more specific early detection. Moreover, the outcome measures used in the study may not cover all aspects of healthcare load during pandemics. The coefficient *r*^FND^ shows correspondences between the nowcasted and observed series of hospitalization incidences over time, and MAPE displays how much the nowcasted incidences deviated as a percentage from the observed incidences. In future studies of COVID-19 hospitalizations, nowcasting the prevalence of hospitalized patients can be considered, which will require considering the length of hospital stay for different categories of COVID-19 patients. Moreover, the study did not use accuracy metrics such as uncertainty bounds around the point predictions because the public health practitioners did not request such bounds. It would have been possible to change the evaluation metrics afterwards, but doing so would have neutralized the prospective evaluation design. In the future, the nowcasting method can be further developed by including uncertainty bounds or probability estimates (*41*). The current approach has at least 2 uncertainties that can be quantified; uncertainty about how many persons with symptoms call the telenursing service, and uncertainty about the proportion of calls for a specific chief complaint that is constituted by COVID-19 cases. Finally, the nowcasting method was intended for use during the initial stage of a pandemic when broad laboratory testing is unavailable. The results are mainly generalizable to other early pandemic settings in which comparable infrastructural resources are available. Generalization of our results and application of the nowcasting method to later pandemic phases, when population-level laboratory testing is available, warrants more research.

We conclude that symptom data regularly recorded in healthcare can be used for local nowcasting of hospital loads during the initial stage of a pandemic when broad laboratory testing still is unavailable. The telenursing chief complaint cough by adult displayed satisfactory nowcasting performance during initial pandemic periods with high community dissemination of COVID-19 (>1.5 hospitalization cases/day/100,000 population). The study also indicates that symptom data should be used with caution for pandemic nowcasting when the novel virus is co-circulating with competing viruses. Our results support local nowcasting of hospitalizations on the basis of regularly recorded syndromic data during the initial stage of a pandemic.

Appendix Additional information about nowcasting of coronavirus disease hospitalizations using syndromic healthcare data, Sweden, 2020.
