# Supplementary figures and images for: Nowcasting (Short-Term Forecasting) of COVID-19 Hospitalizations Using Syndromic Healthcare Data, Sweden, 2020
Source: Emerg Infect Dis. 2022 Mar;28(3):564–71. doi: 10.3201/eid2803.210267 (PMC8888224; doi:10.3201/eid2803.210267)

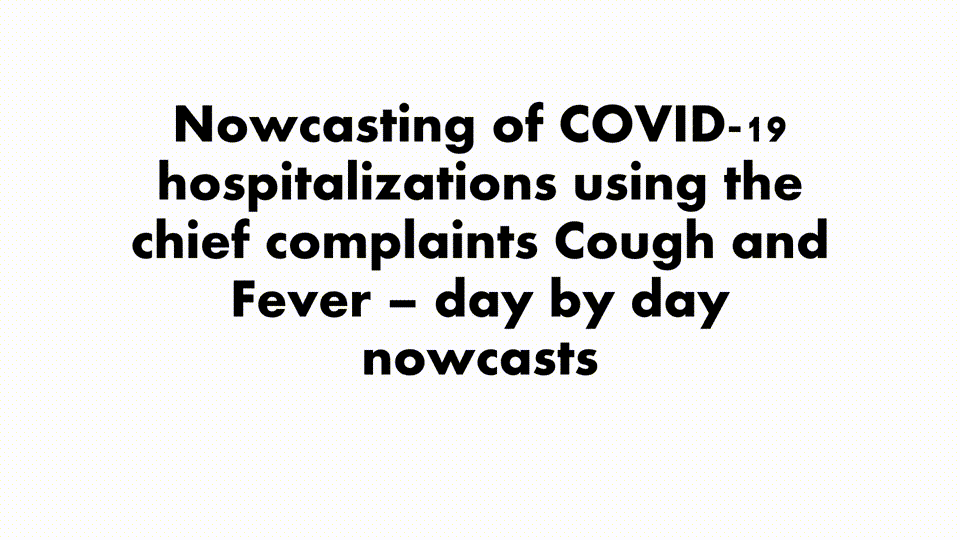

Supplement: Supplementary file 1 [file 21-0267-V.gif]
