# Supplementary material for: Nowcasting (Short-Term Forecasting) of COVID-19 Hospitalizations Using Syndromic Healthcare Data, Sweden, 2020
Source: Emerg Infect Dis. 2022 Mar;28(3):564–71. doi: 10.3201/eid2803.210267 (PMC8888224; doi:10.3201/eid2803.210267)
Supplement: Appendix — Additional information about nowcasting of coronavirus disease hospitalizations using syndromic healthcare data, Sweden, 2020. [file 21-0267-Techapp-s1.pdf]

Article DOI: https://doi.org/10.3201/eid2803.210267

Nowcasting (Short-Term Forecasting) of COVID-19 Hospitalizations Using Syndromic Healthcare Data, Sweden, 2020

Appendix

Method Overview

Data on the chief complaints from calls made by county residents to the Swedish Healthcare Direct service (referred to as telenursing data) were used to nowcast patient hospitalizations due to COVID-19 (referred to as hospitalizations). Calls possibly instigated due to COVID-19 were identified using the chief complaint codes for cough by adult and fever by adult and from the fixed-field terminology register for the national telenursing database (Hälsoläge). Variation in hospitalizations was assumed to reappear with a similar pattern as incidence data for each of these chief complaints, although at a proportional level and with a time lag (delay). The preparatory analyses for the nowcasting aimed at (1) finding which time lag is best in nowcasting hospitalizations for each of the 2 chief complaints and (2) finding the appropriate level of proportional adjustment of telenursing data to be used for nowcasting hospitalizations. Nowcasting of hospitalizations was done in parallel separately for cough by adult or fever by adult, making it possible to compare the ability of these 2 chief complaints to nowcast hospitalizations.

We initially performed retrospective analyses to select the optimal time lag and proportional adjustment for each of the 2 chief complaints. The telenursing data were arranged in 16 time series (the 2 chief complaints with each of 8 possible time lags: 14 to 21 days). To eliminate weekday effects, which both telenursing data and hospitalization data are affected by, all series were smoothed by calculating a 7-day moving average. For each of these series, we assessed Pearson's correlation (r) with hospitalizations. We chose the 2 series (1 each for cough by adult and fever by adult) that had the highest correlation coefficient for nowcasts of patient hospitalizations. We assumed the number of telenursing calls and the number of new hospitalizations to be at different levels but with similar variation patterns, although with

hospitalizations lagging 14–21 days behind telenursing. Suppose that the time lag is 1 day instead of 14–21 days; to get the pattern and timing correct, a nowcast of hospitalizations tomorrow is based on telenursing today. To also get the level right, the level of telenursing today should be adjusted by hospitalizations today divided by telenursing yesterday. The reported nowcasts are based on a corresponding adjustment but with a longer time lag (which is determined as one step in the procedure), and with respect to the fact that weekday effects exist and are not identical for the 2 variables. To adjust for the higher daily rates of telenursing calls compared with the hospitalization rates, we made proportional adjustments of the level of telenursing data by multiplying these data by a ratio. This ratio was calculated by dividing the sum of hospitalizations 0 to 13 days back in time with the sum of telenursing calls over a 14-day interval further back in time at a time distance depending on the resulting best time lag. For instance, if the best time lag was τ days, we calculated the ratio used for adjustment of the level by dividing the sum of hospitalizations 0–13 days back in time by the sum of telenursing calls τ to τ +13 days back in time. The length of the interval should be a multiple of 7 to level out weekday effects and be about the same as the time lag. Therefore, we chose an interval of 14 days.

Thereafter, the hospitalization nowcasting was based on the best time lag (in relation to daily hospitalizations) of the 2 telenursing chief complaints, cough by adult and fever by adult, and was produced every day to guarantee that the nowcasts were always produced using the latest data. When we completed the telenursing data series with data for a new day, we calculated nowcasts of hospitalizations up to τ days (depending on the best time lag) forward in time.

Method Descriptions

2.1 Data-Generating Model

Henceforth “call” is used instead of “telenursing call with chief complaint cough by adult.” Calls $u(t)$ made on day t can be related to hospitalizations on day $y(t + \tau)$, τ days later, through

$$y(t + \tau) = \theta(t, \tau)u(t) + e(t, \tau), \qquad (1)$$

where the scale factor θ can be interpreted as “hospitalizations in τ days per calls today,” and $e(t)$ is a random component.

2.2 Assumptions

From early reporting, it is assumed that the pattern/variation of calls day 1 to $t - \tau^o$ will reappear in the pattern of hospitalizations on day $1 + \tau^o$ to t, where $14 \leq \tau^o \leq 21$. If we assume that this relation holds for at least another τ^o days and that τ^o is known and time-invariant, we can fix $\tau = \tau^o$ and define $\theta^0(t) := \theta(t,\tau^o)$, $e^o(t,\tau^o) := e(t)$ in (1) to obtain

$$y(t + \tau^o) = \theta^0(t)u(t) + e^o(t). \qquad (2)$$

If, furthermore, we can assume that the influence of random effects $e^o(t)$ are small on $y(t + \tau^o)$ in (2), and that $\theta^0(t)$ varies smoothly enough, then it may be feasible to first form an estimate $\hat{\tau}$ of τ^o and subsequently use it together with historic u,y to form an estimate $\widehat{\theta}$ of θ^0.

2.3 Nowcasting

Daily values of $u(k)$ and $y(k)$ are assumed available for each day $k \geq 1$ up to and including the current day t (Appendix Table). On each day $t > k_{\max}$, the model produces a sequence of nowcasts for the subsequent days $t+1,...,t+\hat{\tau}(t)$ according to

$$\hat{y}_f(t + k) = \widehat{\theta}(t)u_f(t + k - \hat{\tau}(t)), \; k = 1,...,\hat{\tau}(t). \qquad (3)$$

The subscript f in u_f of (3) denotes a 7-day moving average filtration

$$u_f(k) = \frac{1}{7}\sum_{m=0}^{6} u(k-m)$$

2 , (4)

and we define y_f analogously.

For each day $t > k_{\max}$, and time shift $k_{\min} \leq k \leq k_{\max}$, we define the windowed time series

$$u_k(t) = [u_f(7),...,u_f(t - k)], \qquad (5)$$

$$y_k(t) = [y_f(k + 7),...,y_f(t)]. \qquad (6)$$

The estimate $\hat{\tau}(t)$ of the time lag parameter τ^o is adaptively updated through maximizing the correlation coefficient between $u_k(t)$ and $y_k(t)$ over the considered lags $k_{\min} \leq k \leq k_{\max}$:

$$\hat{\tau}(t) = \underset{k = k\min,...,k\max}{\operatorname{argmax}} \; r(u_k(t), y_k(t)). \qquad (7)$$

The adjustment ratio $\widehat{\theta}$ of (3) is also adaptively updated:

$$\hat{\theta}(t) = \frac{\sum_{m=0}^{M-1} y(t-m)}{\sum_{m=0}^{M-1} u(t-m-\hat{\tau}(t))}. \qquad (8)$$

We note that k_{min} has been chosen as a multiple of 7 days to eliminate weekday effects (Appendix Table). A consequence is that the algorithm is applicable as long as 7-day moving averages of u and y are available up to and including the day that a nowcast is computed.

Appendix Table. Signals and variables used within the nowcasting algorithm for forecasting coronavirus hospitalizations by symptom data, Sweden, February–June 2020*

Variable	Meaning
Daily per-region time series of reported, unfiltered values	
u	Telenursing calls
y	COVID-19 hospitalizations
$\hat{y}$	Nowcasted (i.e., predicted) hospitalizations
Time-varying nowcasting model parameters	
$\hat{\tau}$	Estimate of the time lag parameter τ^0 (Appendix Section 2.2)
$\hat{\theta}$	Estimate of the time-varying adjustment ratio (i.e., scale factor) parameter θ (Appendix Section 2.2)
Constant nowcasting model parameters	
$k_{min} = 2 \cdot 7$	Lower bound for $\hat{\tau}$ (2 weeks)
$k_{max} = 3 \cdot 7$	Upper bound for $\hat{\tau}$ (3 weeks)
$M = 2 \cdot 7$	Window width used to obtain $\hat{\theta}$ (2 weeks)

*COVID-19, coronavirus disease.
